# Supplementary figures and images for: Tethered Exosomes Containing the Matrix Metalloproteinase MT1‐MMP Contribute to Extracellular Matrix Degradation
Source: J Extracell Vesicles. 2025 Jul 24;14(7):e70122. doi: 10.1002/jev2.70122 (PMC12287793; doi:10.1002/jev2.70122)

Figure S1.

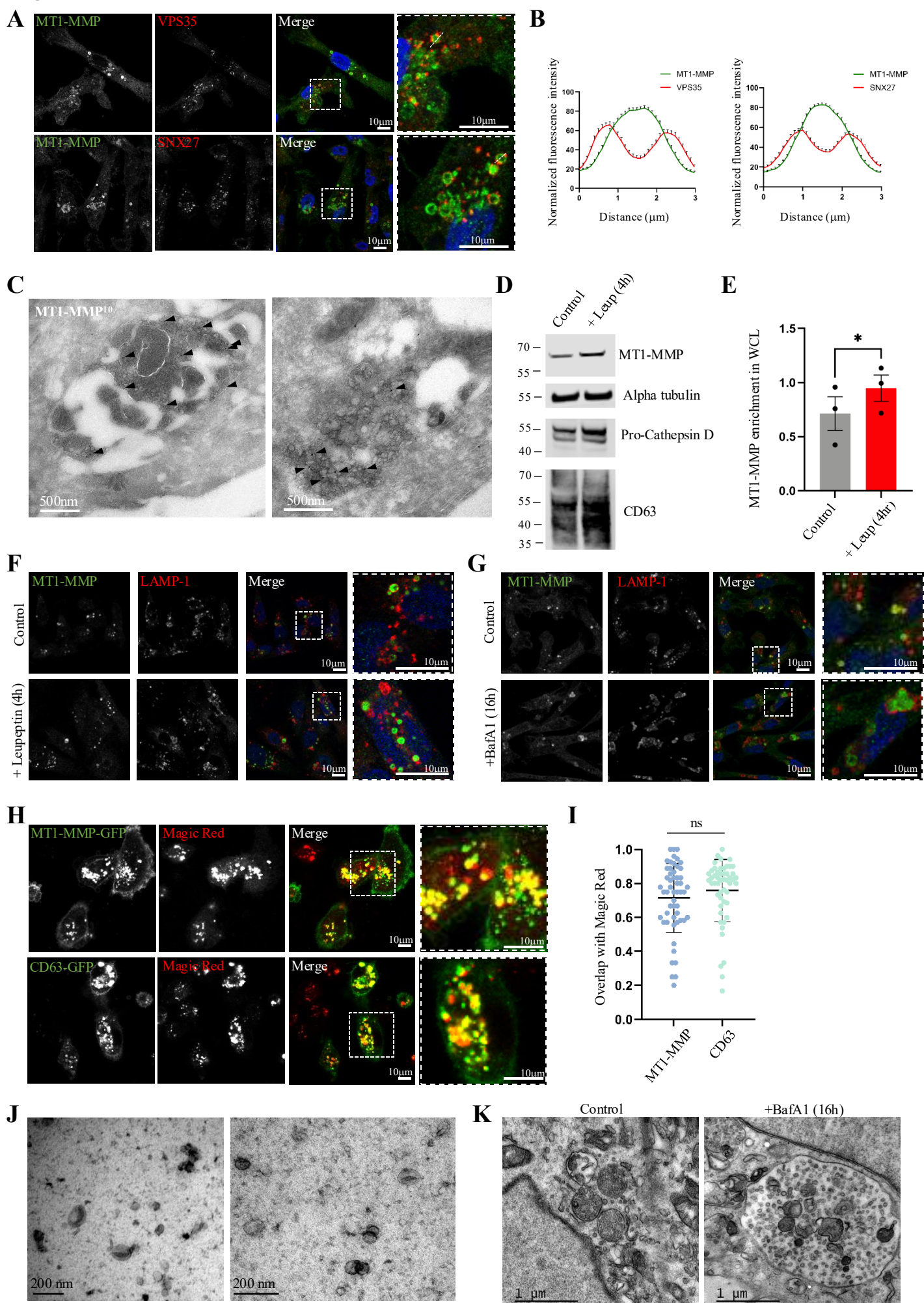

Supplement: Supplementary file 1 — Supporting Fig.1: Supplemental data related to Figure 1 . (A) Confocal analysis of endogenous MT1‐MMP localization with VPS35 or SNX27 in MDA‐MB‐231 WT cells. Scale bars = 10 µm. Dashed lines represent the 3 µm drawn to perform the line scan analysis in (B). (B) Line scan analysis of MT1‐MMP with VPS35 or SNX27 fluorescence intensity in endosomes. n ≥ 81 endosomes, error bars represent SEM. (C) Further examples of immuno EM micrographs of MDA‐MB‐231 WT cells labelled with MT1‐MMP and PAG 10 nm. Scale bar = 500 nm. (D) Western blotting analysis of WCL from MDA‐MB‐231 cells control or treated with Leupeptin for 4 h. (E) Enrichment of MT1‐MMP in WCL from MDA‐MB‐231 cells control or treated with Leupeptin for 4 h. n = 3 biologically independent experiments, paired t test, * p = 0.0204. (F) Confocal analysis of endogenous MT1‐MMP localization with LAMP‐1 in MDA‐MB‐231 cells control or treated with Leupeptin for 4 h. Scale bars = 10 µm. (G) Confocal analysis of endogenous MT1‐MMP localization with LAMP‐1 in MDA‐MB‐231 cells control or treated with BafA1 for 16 h. Scale bars = 10 µm. (H) Confocal analysis of MT1‐MMP‐GFP or CD63‐GFP localization with active Cathepsin B dye Magic Red in MDA‐MB‐231 WT cells. Scale bars = 10 µm. (I) Overlap ratio of MT1‐MMP‐GFP or CD63‐GFP with Magic Red (mean ± SD). n = 51 for CD63, 53 for MT1‐MMP, cells from three biologically independent experiments, unpaired t test. (J) EM micrograph of sEVs isolated from MDA‐MB‐231 WT cells. Scale bars = 200 nm. (K) EM micrograph of MDA‐MB‐231 WT cells treated with DMSO or BafA1 for 16 h. Scale bars = 1 µm. [file JEV2-14-e70122-s003.pdf]

Figure S2.

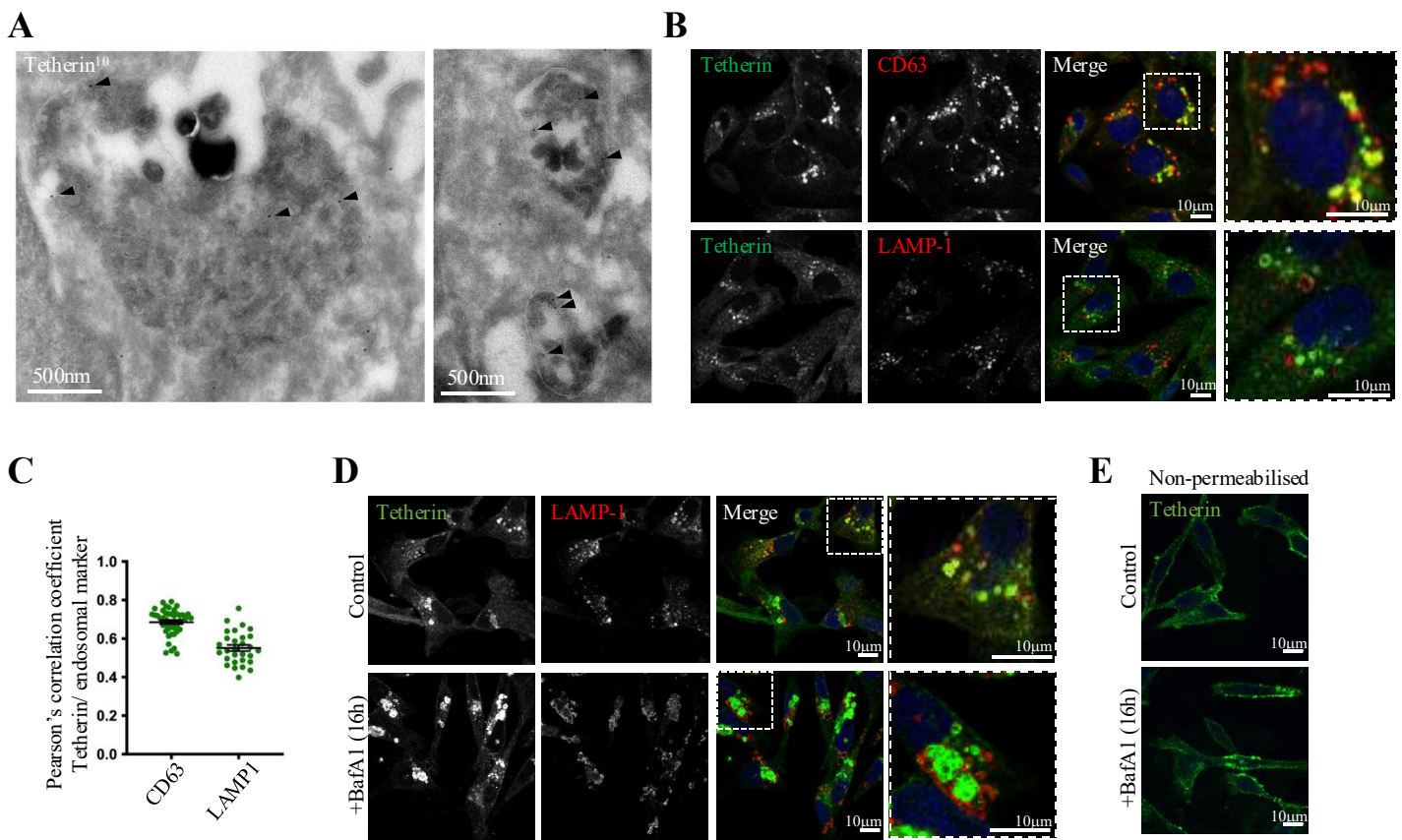

Supplement: Supplementary file 2 — Supporting Fig.2: Supplemental data related to Figure 3 . (A) Immuno EM micrographs of MDA‐MB‐231 WT cells labelled with anti‐tetherin antibody and PAG 10 nm. Scale bar = 500 nm. (B) Confocal analysis of tetherin localization with CD63 or LAMP‐1 in MDA‐MB‐231 cells. Scale bars = 10 µm. (C) Pearson's correlation coefficient of tetherin and CD63 or LAMP‐1. n = 44 for CD63, 29 for LAMP‐1, cells from two biologically independent experiments, error bars represent SEM. (D) Confocal analysis of tetherin localization with LAMP‐1 in MDA‐MB‐231 cells control or treated with BafA1 for 16 h. Scale bars = 10 µm. (E) Surface staining of MDA‐MB‐231 WT cells labelled with tetherin. Scale bars = 10 µm. [file JEV2-14-e70122-s004.pdf]

Figure S3.

**A**

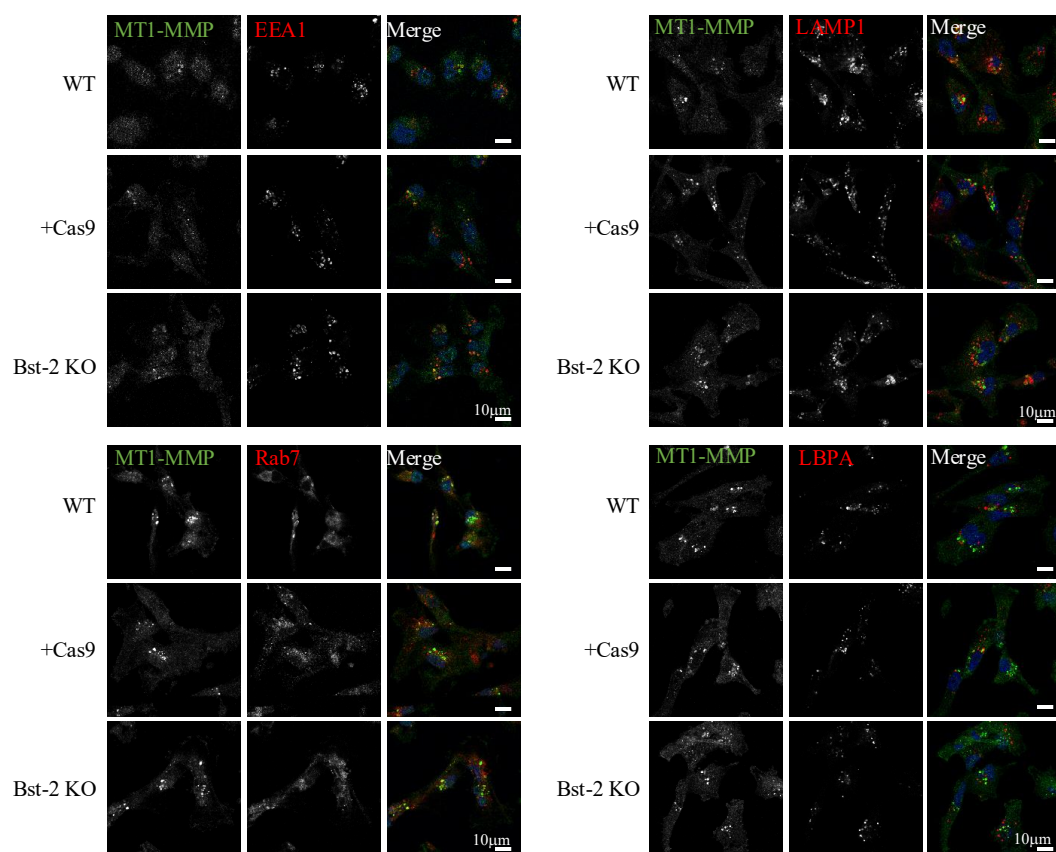

**B**

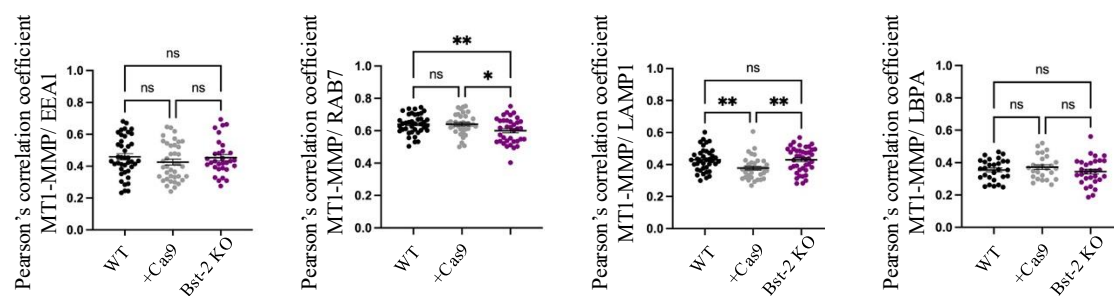

**C**

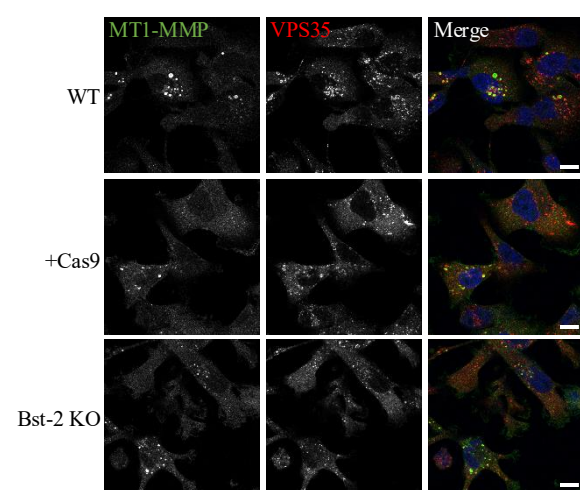

**D**

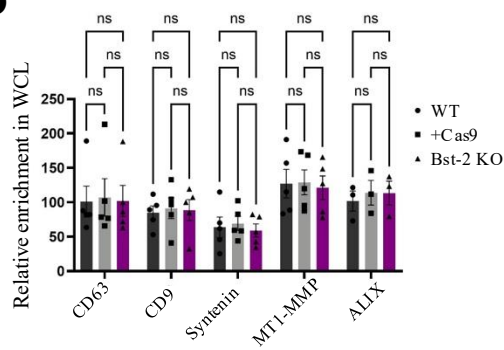

**E**

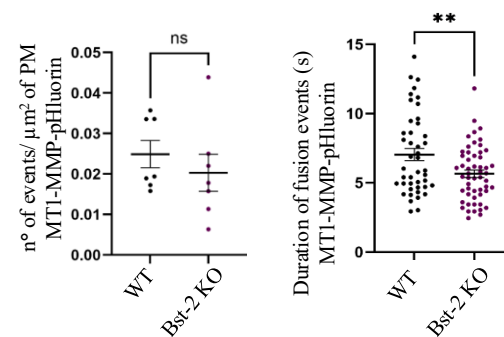

**F**

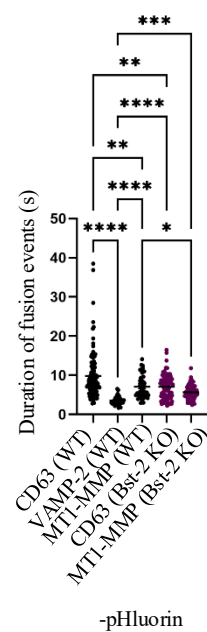

Supplement: Supplementary file 3 — Supporting Fig.3: Supplemental data related to Figure 4 . (A) Confocal analysis of endogenous MT1‐MMP localization with different endosome/lysosome markers in MDA‐MB‐231 cell lines. Scale bars = 10 µm. (B) Pearson's correlation coefficient of MT1‐MMP and endosome/lysosome markers. n ≥ 25 cells from three biologically independent experiments, mean ± SEM, ordinary one‐way ANOVA, * p = 0.0111 and ** p = 0.0097 for RAB7, ** p = 0.0025 and ** p = 0.0022 for LAMP‐1. (C) Confocal analysis of endogenous MT1‐MMP localization with VPS35 in MDA‐MB‐231 cell lines. Scale bars = 10 µm. (D) Relative enrichment of the protein content in WCL from MDA‐MB‐231 cell lines (mean ± SEM). n = 5 biologically independent experiments, two‐way ANOVA. (E) Fusion activity of MT1‐MMP‐pHluorin. n = 7 cell fusion events per reporter from three biologically independent experiments, t‐test with Welch correction. Signal duration of fusion events of MT1‐MMP‐pHluorin. n = for WT, 54 for Bst‐2 KO, 44 fusion events per reporter from three biologically independent experiments, ordinary one‐way ANOVA, ** p = 0.0077. (F) Signal durations of all pHlourin data, from CD63‐pHlourin (WT), VAMP‐2‐pHlourin (WT), MT1‐MMP‐pHlourin (WT), CD63‐pHlourin (Bst2 KO), MT1‐MMP‐pHlourin (Bst2 KO). Mean± SEM, Kruskal–Wallis test, * p = 0.0412, ** p = 0.0068 and 0.0015, *** p = 0.0003, **** p < 0.0001. [file JEV2-14-e70122-s005.pdf]

Figure S4.

**A**

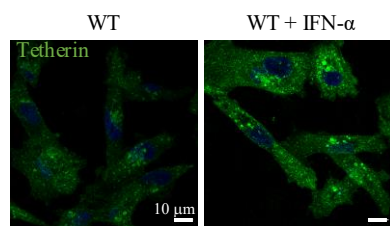

**B**

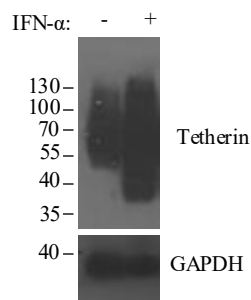

**C**

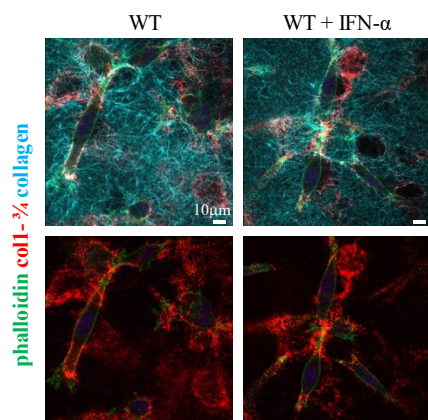

**D**

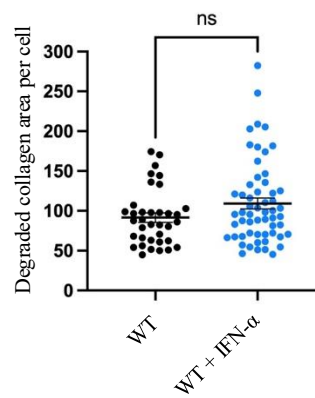

**E**

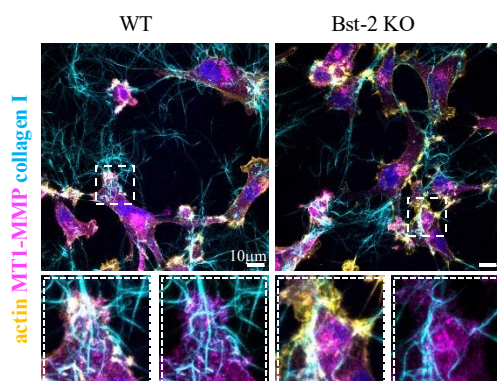

Supplement: Supplementary file 4 — Supporting Fig.4: Supplemental data related to Figure 5 . (A) Confocal analysis of MDA‐MB‐231 WT control or treated with IFN‐α or MDA‐MB‐231 WT + Bst‐2‐HA stained for tetherin. Scale bars = 10 µm. (B) Western blotting analysis of tetherin expression in MDA‐MB‐231 cell lines control or treated with IFN‐α. (C) Confocal analysis of collagen degradation assay. MDA‐MB‐231 WT control or treated with IFN‐α were embedded in 3D fluorescent collagen gels, incubated for 24 h, fixed and stained with phalloidin and for the cleaved collagen new epitope using anti‐Col‐3/4 antibody. Scale bars = 10 µm. (D) Quantification of pericellular collagen degradation expressed as average degraded collagen area per cell by MDA‐MB‐231 WT control or treated with IFN‐α (mean ± SEM). n = 36 for WT, 57 for WT+ IFN‐α confocal images (≥ 300 cells) from three biologically independent experiments, Kruskal–Wallis test. (E) Confocal analysis of invadopodia formation assay. Cells were plated on a thin layer of fluorescent collagen, incubated for 60 min, fixed, and stained with MT1‐MMP and phalloidin. Magnification shows the presence of MT1‐MMP in proximity of linear invadopodia along collagen fibres. Scale bars = 10 µm. [file JEV2-14-e70122-s006.pdf]
